# PubChem BioAssays 1063: A Poorly Exploited Source of New Antileishmanial Compounds

**DOI:** 10.1155/japr/6338486

**Published:** 2025-06-27

**Authors:** Sergio Sifontes-Rodríguez, Susana Meneses-Gómez, Alma Reyna Escalona-Montaño, Daniel Andrés Sánchez-Almaraz, Ofelia Pérez-Olvera, Aranza Regina Cañón Rosas, Pedro Zuriel Cruz Bautista, María Magdalena Aguirre-García

**Affiliations:** ^1^Faculty of Medicine, National Autonomous University of Mexico, UNAM-INC Research Unit, National Institute of Cardiology “Ignacio Chávez”, Mexico City, Mexico; ^2^Faculty of Pharmacy, “Marta Abreu” Central University of Las Villas, Santa Clara, Cuba

**Keywords:** AID 1063, BALB/c mice, cutaneous leishmaniasis, intracellular amastigotes, *Leishmania amazonensis*, *Leishmania infantum*, *Leishmania major*, *Leishmania mexicana*, promastigotes

## Abstract

PubChem Bioassays (AID 1063) reported the in vitro testing of 196,141 compounds against *Leishmania major* promastigotes. Although these results have been publicly available since 2008, limited efforts on further testing of some of these compounds has been published. The aim of the present work was selecting a small set of compounds that were highly active in that primary assay and assessing their antileishmanial activity in vitro and in vivo. Selected compounds were 100% active in the primary assay at 10 *μ*M, were not theoretically toxic, did not have structural features of pan assay interfering substances, had positive druglikeness, and were not cytotoxic, and their activity rate in previous assays reported in PubChem Bioassays was under 5%. Seven commercially available compounds were purchased and tested against *L. major*, *Leishmania mexicana*, *Leishmania amazonensis*, and *Leishmania infantum* promastigotes; in mouse peritoneal macrophages (cytotoxicity); and against *L. mexicana* intracellular amastigotes. Eventually, four compounds with appropriate selectivity and high activity against *L. mexicana* amastigotes were tested by intralesional route (1%, 20 *μ*L) in a mouse model of cutaneous leishmaniasis. Four compounds were active (IC_50_ < 10* μ*M) against the promastigote stage of the four *Leishmania* species tested. These four compounds were also active (IC_50_ < 10* μ*M) in vitro against intracellular amastigotes and in vivo in mice experimentally infected with *L. mexicana*. Results demonstrated the potential of these compounds as antileishmanials and the high, unexploited potential of AID 1063 as a source of new antileishmanial agents.

## 1. Introduction

Leishmaniasis is a vector-borne infection caused by nearly 20 kinetoplastid protozoan species in the genera *Leishmania* and *Endotrypanum*. It occurs in various clinical forms, the most frequent being cutaneous leishmaniasis, visceral leishmaniasis, and mucocutaneous leishmaniasis [1]. Leishmaniasis is prevalent in low-income and developing countries. Over 12 million people are infected with the causative agents, and 350 million are at risk of infection, making leishmaniasis the third most important neglected tropical disease [2].

Current drugs for the treatment of leishmaniasis are pentavalent antimonials (sodium stibogluconate and meglumine antimoniate), amphotericin B (amphotericin B deoxycholate and liposomal amphotericin B), miltefosine, paromomycin, and pentamidine. However, in general, they are limited by one or more of the following issues: toxicity, variable efficacy, parasite resistance, parenteral administration, lack of availability, and cost of treatment and hospitalization [3]. Consequently, for years, there has been a call to prioritize the search for better new drugs for the treatment of leishmaniasis. Academy, industry, government, and nongovernment organizations have collaborated, and many scientific results have accumulated over the years. Previously, small sets of compounds were usually tested, but the advent of high-throughput screening (HTS) technology has enabled the screening of collections of thousands [4–6] and millions of compounds [7, 8] in recent times. For a detailed revision of the topic, the reader is referred to the review by Martin et al. [9].

The largest collections tested so far included 1.8 million compounds from GlaxoSmithKline [8] and 2.9 million compounds from Novartis [7]. The GlaxoSmithKline HTS screening collection was tested against *Leishmania (Leishmania) donovani* axenic amastigotes. The active compounds found in the primary screening were further tested and selected, and eventually, 351 noncytotoxic compounds active against intracellular amastigotes were identified. A structurally diverse set of 192 out of them was selected (Leish-Box), and physical samples were made available to collaborators upon request [8].

The 2.9 million compound collection of Novartis was similarly screened against *L. donovani* axenic amastigotes, and an azabenzoxazole hit (GNF5343) was identified and later optimized (compound GNF 6702) in terms of potency (400-fold increase), selectivity, safety, and bioavailability. Compound GNF 6702 also resulted active against *Trypanosoma cruzi* and *Trypanosoma brucei* in vitro and in vivo [7]. Unfortunately, primary data of structures and associated activities resulting from these two HTS campaigns are not publicly available.

Another outstanding HTS campaign searching for antileishmanial compounds was conducted by Sharlow et al. [4] at the University of Pittsburgh Molecular Library Screening Center. A total of 196,141 compounds were tested against *Leishmania (Leishmania) major* promastigotes at 10 *μ*M, 17,620 of which were active. A selection of 1121 was further tested at 1 *μ*M resulting in 146 active compounds, 93 of which had 50% inhibitory concentrations under 1 *μ*M. Sharlow et al. [4] found 15 representative chemotypes with antileishmanial activity and demonstrated the in vivo activity of one compound (disulfiram) in an experimental model of cutaneous leishmaniasis. Noticeably, the experimental outcomes and structures of all the tested compounds have been publicly available since 2008 in the PubChem Bioassays database AID 1063 [10]. However, there are few references to the use of this information, particularly for the subsequent development of compounds previously found to be active.

In the present paper, we report the rational selection, as well as in vitro and in vivo testing, of seven compounds previously found active by Sharlow et al. [4] at 10 *μ*M, but not tested at lower concentrations. We demonstrate the high, unexploited potential of the information disclosed in AID 1063 for developing new antileishmanial compounds.

## 2. Material and Methods

### 2.1. Selection of Compounds

The two major datasets associated to the project entitled “Identification and Optimization of Small Molecule Inhibitors of *Leishmania* Parasite Growth and Viability” conducted at the University of Pittsburgh Molecular Library Screening Center by using HTS technology were used. These datasets are publicly available under the PubChem Bioassays identifiers AID 1063 and AID 1258 [10, 11]. Dataset AID 1063 includes 196,141 compounds tested a 10 *μ*M against *L. major* promastigotes, of which 17,620 were active, that is, reduced parasite growth under 50% of that of untreated cultures. A selection of 1121 of such active compounds was later tested at 1 *μ*M, 146 of which also resulted active at that lower concentration [11].

Results of compounds reported in both datasets, that is, those tested at 10 *μ*M and then at 1 *μ*M, were merged into a single Excel file. Compounds with inconclusive outcomes at 1 *μ*M were excluded. The compounds were grouped according to their activity score (from 20 to 40, which corresponds to 50%–100% growth inhibition at 10 *μ*M), and the percentage of active compounds at 1 *μ*M was then estimated for each group of activity score. Finally, an analysis of the relationship between growth inhibition at 10 *μ*M and the outcome (positivity rate) at 1 *μ*M was carried out.

The SMILES of the 1698 compounds that caused 100% growth inhibition at 10 *μ*M and that were not tested at 1 *μ*M were loaded into Osiris DataWarrior software [12] for the prediction of their toxicological properties. These included the mutagenic, tumorigenic, and irritant potential as well as the potential of causing reproductive deleterious effects. Additionally, the druglikeness and drugscore were calculated. Compounds with a positive prediction of any of the toxicological properties or with negative druglikeness were excluded. The drugscore shows the overall likelihood of a compound meeting the criteria to become a drug candidate in light of the druglikeness, cLogP, solubility, molecular weight, and toxicity hazards [13] and was used to rank the final selection of compounds (see below).

Afterwards, a substructural search for molecular features of pan assay interfering substances (PAINSs) was conducted by using Osiris DataWarrior. For this, the 44 structures established by Dahlin and Walters [14] were used as patterns. Compounds containing any of such fragments were eliminated because of their probability of failure due to lack of specificity. The remaining compounds were grouped in structural clusters and ranked in each cluster according to their drugscore. A set of 30 structurally different compounds (belonging to different clusters) was chosen to check their commercial availability, reported cytotoxicity, and activity, in special, previously described antileishmanial activity. For cytotoxicity and activity, the results available in PubChem Bioassays records were downloaded for each of the compounds. Then, the percent of assays with positive outcomes (activity rate) was calculated, and compounds with an activity rate over 5% were discarded, as well as any compound with a report of cytotoxicity (no matter the cell line) at concentrations under 10 *μ*M. Previous reports of antileishmanial activity were explored in PubMed and Google Scholar. Eventually, seven compounds were purchased and tested for their potential as antileishmanial drugs.

#### 2.1.1. Test Compounds

Compounds 417352 (MCULE-7016129622), 706914 (MCULE-3572497728), 314212 (MCULE-4968705855), 4362222 (MCULE-9576722559), 662265 (MCULE-5064189776), 15945681 (MCULE-8014360482), and 3103717 (MCULE-3271853615) were purchased from Mcule (https://mcule.com/) with guaranteed purity over 90%. Stock solutions at 10 mg/mL in dimethyl sulfoxide (DMSO, Sigma-Aldrich, St. Louis, MO, United States) were made and stored at 4°C in the dark until used. Proper dilutions in DMSO were prepared according to the assay. The maximum concentration of DMSO in any of the culture media was 0.5%.

### 2.2. Parasites

*Leishmania (Leishmania) amazonensis* MHOM/BR/77/LTB0016 reference strain was kindly donated by the Department of Immunology of Fundação Oswaldo Cruz (Fiocruz), Brazil. *Leishmania (Leishmania) mexicana* MNYC/BZ/62/M379 and *L. major* MHOM/IL/81/Friedlin reference strains were kindly donated by Paul A. Bates, Division of Biomedical and Life Sciences, Faculty of Health and Medicine, Lancaster University, United Kingdom. *Leishmania (Leishmania) infantum* MHOM/FR/78/LEM75 was kindly donated by the Department of Parasitology, Faculty of Pharmacy, Universidad Complutense de Madrid, Spain. The promastigotes were cultivated at 26°C in Medium 199 (Gibco, Grand Island, NY, United States) supplemented with 10% heat-inactivated (56°C, 30 min) fetal bovine serum (Gibco, Grand Island, NY, United States) and antibiotics (200 IU penicillin and 200 *μ*g/mL streptomycin). Exponential multiplication of the promastigotes of the four species was attained by passages every 3–4 days. *L. mexicana* axenic amastigotes were cultured in Schneider's Insect Medium (Sigma-Aldrich, St. Louis, MO, United States) at pH 5.4, supplemented with 20% heat-inactivated fetal bovine serum and antibiotics (as above).

### 2.3. Animals

Female, 18–20 g, 8–10 weeks old BALB/c mice were supplied by the laboratory animal breeding facility of the Faculty of Medicine, National Autonomous University of México. They were maintained under controlled environmental conditions (room temperature 22°C–25°C, relative humidity 60%–65%, light cycle 12 h light–12 h dark) and were handled by qualified personnel. At the end of studies, mice were sacrificed by CO_2_ inhalation. The experimental protocol was approved by the Ethics in Research Committee and the Internal Committee on the Care and Use of Lab Animals (Code CICUAL 004-CIC-2019). All the experiments were conducted in accordance with the Mexican Regulation [15] and the Institute of Laboratory Animal Research Guide [16] for the care and use of laboratory animals.

### 2.4. Cytotoxicity

The cytotoxicity of the test compounds was assessed in primary cultures of mouse peritoneal macrophages as reported elsewhere, with minor modifications [17]. Briefly, BALB/c mice were sacrificed by CO_2_ inhalation, and their peritoneal macrophages were collected by washing the abdominal cavity with cold RPMI 1640 (Gibco, Paisley, Scotland, United Kingdom) culture medium supplemented with antibiotics (sodium penicillin 200 IU and streptomycin 200 *μ*g/mL) and 10% fetal bovine serum. The macrophages were distributed in 96-well culture plates at 10^5^ cells/well. After 4 h incubation at 37°C and 5% CO_2_, the culture medium was replaced by fresh medium containing the test compounds, and the plates were incubated for another 48 h. Afterwards, 20 *μ*L alamar blue (Thermo Fisher Scientific, Oregon, United States) was added per well. Incubation was continued for another 6–8 h at 37°C and 5% CO_2_ to measure the fluorescence of the reduced dye in a plate reader (excitation: 485 nm, emission: 590, scaling factor: 10/10, Fluoroskan Ascent FL 2.5 plate reader—Thermo Labsystems, Waltham, MA, United States). Fifty percent cytotoxic concentrations (CC_50_) were estimated by nonlinear fitting to the Emax sigmoid model [18]. Each concentration was tested in triplicate, and each compound was tested in three independent studies.

### 2.5. In Vitro Activity Against Promastigotes

The growth inhibition assay of promastigotes was conducted according to Bodley et al.'s procedure [19], with minor modifications. Briefly, 96-well culture plates were seeded with 100 *μ*L culture medium 199 containing 5 × 10^5^ log-phase promastigotes and 100 *μ*L of twofold serial dilutions of the test compounds in culture medium. The plates were sealed with parafilm and incubated for 48 h at 26°C. Negative control wells contained promastigotes and DMSO at 0.5%, and positive wells contained promastigotes and 2.7 *μ*M amphotericin B deoxycholate (Gibco, Grand Island, NY, United States). A series of amphotericin B concentrations was also tested in parallel to estimate its IC_50_ against promastigotes of the three *Leishmania* strains. After the incubation period, 30 *μ*L of 3 mg/mL *p*-nitro-phenyl-phosphate (*p*NPP) (Sigma-Aldrich) in sodium acetate (pH 5.5, Sigma-Aldrich) and 1% Triton X-100 (Sigma-Aldrich) were added to each well. Plates were incubated for other 3 h at 37°C, and 50 *μ*L 0.25 M NaOH (Sigma-Aldrich) was added to change all the produced p-nitro-phenol to its yellow-colored form. Absorbance was then read in a Biotek Epoch microplate reader (Agilent, Santa Clara, CA, United States) at 405 nm. Half-maximal inhibitory concentrations (IC_50_) were estimated by nonlinear fitting of absorbance versus compound concentration to the sigmoid Emax equation [18]. Each drug concentration was tested in quadruplicate, and the assay was repeated three times.

### 2.6. In Vitro Activity Against Intracellular Amastigotes

The activity of the test compounds against *L. mexicana* intracellular amastigotes was carried out by the transformation and proliferation assay as reported elsewhere [20]. Briefly, mouse peritoneal macrophages were obtained by peritoneal lavage of BALB/c mice and cultivated in RPMI 1640 medium supplemented with fetal bovine serum and antibiotics as described above. The cells were seeded in 96-well culture plates at 10^5^ cells/well (100 *μ*L) and incubated at 37°C and 5% CO_2_ for 2 h. Then, macrophages were infected with *L. mexicana* axenic amastigotes at a rate of three parasites per host cell. After overnight incubation at 37°C and 5% CO_2_, noninternalized amastigotes and nonadhered macrophages were removed by the elimination of the culture medium and washing with phosphate buffer solution (PBS). Fresh RPMI 1640 medium containing serial dilutions of the test compounds was then added. Amphotericin B was tested in parallel at concentrations from 1.35 to 0.021 *μ*M. Four replicates of each drug concentration were tested in every assay. Plates were incubated for 48 h at 37°C and 5% CO_2_. Afterwards, the culture medium was discarded, replaced by medium 199, and the plates were incubated for another 72 h at 26°C to allow surviving amastigotes to transform into promastigotes and replicate. *p*NPP was then added to each well as described in the promastigote assay, and the IC_50_ values were estimated as previously indicated.

### 2.7. In Vivo Activity

Mice were infected by intradermal injection in the footpads with 10^7^ stationary-phase *L. mexicana* promastigotes. Two weeks later, mice were randomly allocated in seven groups of 6–7 mice each. Four groups were treated intralesionally every 3 days (five doses) with 20 *μ*L of any of the test compounds at 10 mg/mL in DMSO. Positive control mice were treated with 7.5 mg/kg amphotericin B deoxycholate every 48 h for 14 days [21], vehicle control mice were treated with 20 *μ*L DMSO every 3 days (five doses), and negative controls did not receive any treatment at all. The dorso-plantar thickness of the rear limbs was weekly measured from the beginning of treatment (14 days postinfection) to 2 weeks after the end of treatment. Feet were measured with a Vernier caliper, and lesion sizes were calculated by subtracting the value of the noninfected limb (left) to that of the infected one (right).

Two weeks after the end of treatment, mice were weighed, sacrificed by CO_2_ inhalation, and necropsied. The weights of the heart, liver, spleen, and left and right kidneys were determined. Both rear limbs were excised at the level of the tibiotarsal joint and weighed to calculate, by difference, the weight of the lesions. The number of parasites in the lesions was estimated by using the limiting dilution assay technique [22].

## 3. Results

### 3.1. Selection of Compounds

**Figure 1:**
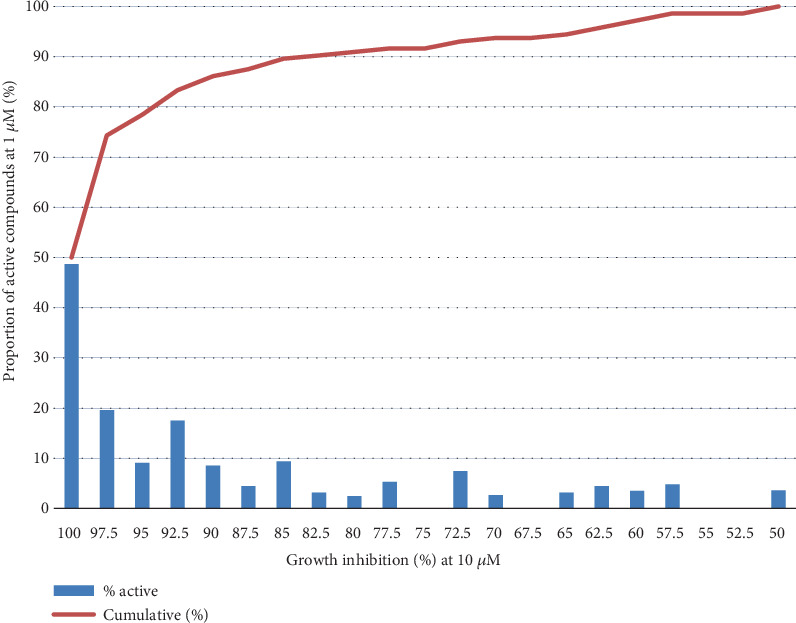
Correlation of activity against *L. major* promastigotes at 10 and at 1 *μ*M. Figure shows the correlation of growth inhibition (percent) induced by a compound in the primary assay at 10 *μ*M [10] with its probability (percent) of being active (*g*rowth inhibition > 50%) once tested in the secondary assay at 1 *μ*M [11].

When comparing the results of the compounds tested by Sharlow et al. [4] at 10 and 1 *μ*M, a close relationship between the growth inhibition elicited by the compounds at 10 *μ*M and the probability of being active (growth inhibition over 50%) at 1 *μ*M was observed (Figure 1). Near 50% of the compounds that produced 100% inhibition at 10 *μ*M were also active at 1 *μ*M. Noticeably, 86% of the compounds that were active at 1 *μ*M were those that showed over 90% growth inhibition at 1 *μ*M. On that basis, a high likelihood of being active at nanomolar concentrations was expected from compounds that showed the highest inhibition at 10 *μ*M.

A total of 1846 compounds produced 100% inhibition at 10 *μ*M, but only 148 of them were further tested at 1 *μ*M. Thus, the remaining 1698 compounds that caused 100% growth inhibition of *L. major* promastigotes at 10 *μ*M and were not tested at 1 *μ*M were selected for the purpose of the present work.

Structure-based estimates of toxicity identified a group of likely toxic compounds (Table 1). Some of them had the potential for more than one kind of toxicity, resulting in 1010 compounds that were theoretically safe regarding the toxicological properties estimations implemented in Osiris DataWarrior software [23].

The PAINS substructural feature search flagged 178 compounds. The commonest PAINS substructures were pyrocatechol (94 compounds), (2E,5E)-hepta-2,5-dien-4-one (30 compounds), and hydroquinone (27 compounds). The presence of PAINS substructures suggests a high potential for the compound to interfere with biological assays. Not every compound containing one of these substructures will be an interference compound, but it has been advised to proceed carefully if an active compound contains one of these substructures [14]. Therefore, all the compounds that were detected as containing a PAINS substructure were removed, resulting so far in 834 highly active, potentially safe and specific compounds. Of these, 401 had positive drug-likeness; that is, they had a chemical structure that was more like pharmaceuticals than nonpharmaceuticals [24, 25].

**Figure 2:**
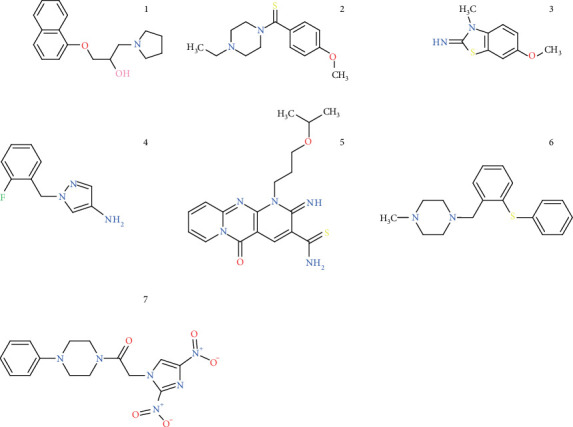
Chemical structure of the test compounds. Selected compounds caused 100% inhibition at 10 *μ*M [10], were theoretically safe, structurally drug-like, noncytotoxic (according to reported PubChem Bioassays), potentially specific (non-pan assay interfering substances and had low positivity rate in PubChem Bioassays), and commercially available.

DataWarrior generated 285 structural clusters of one to eight compounds. Notably, 215 clusters (75%) were singlets, indicating a wide structural diversity among the compounds. At this point, the compounds were sorted according to their drugscore, and the first 30 compounds belonging to different clusters were chosen. Then, considering cytotoxicity, positivity rate, and commercial availability, the selection was progressively narrowed to the final list of seven compounds (Figure 2). All of them have been reported in over 700 biological tests in PubChem Bioassays with a positivity rate from 0.6% to 4.6%, and none of them have been reported as cytotoxic (information available at 10.5281/zenodo.10084616).

The physicochemical properties of the compounds (Table 2) show that all but Compound 7 (which has more than 10 hydrogen acceptor atoms), fulfil Lipinski's rule of five [26] suggesting an expected proper oral bioavailability. Moreover, they have appropriate drug-likeness (positive values: over 1.4) and drugscore (relatively close to 1: from 0.83 to 0.93).

### 3.2. In Vitro Studies

In vitro studies (Table 3) revealed that Compounds 1–3 were poorly active against the promastigote and amastigote stages of *Leishmania* spp. and were also noncytotoxic for mouse peritoneal macrophages (CC_50_ > 100* μ*M). That lack of antileishmanial activity was unexpected since these compounds were reported as highly active at 10 *μ*M against *L. major* promastigotes [4]; therefore, IC_50_ values under or close to 10 *μ*M were expected, at least against *L. major* promastigotes. Considering that the species and strain were the same in both works, such disagreement could be either due to differences in the test conditions, the source of the compounds, or, in the worst case (which we consider less likely due to the assay automation), to annotation errors.

In turn, the remaining four compounds showed a high to moderate degree of antileishmanial activity and CC_50_ values from near 25–170 *μ*M. In particular, Compounds 5 and 7 were highly active and specific (selectivity indices over 100). The lack of cytotoxicity (CC_50_ > 20* μ*M) was expected, considering the previous search conducted in PubChem Bioassays and the selection of compounds with no previous reports of cytotoxicity. On the basis of these results, Compounds 4, 5, 6, and 7 were tested in an animal model of experimental cutaneous leishmaniasis. IC_50_ values of amphotericin B against the promastigote and amastigote stages of *Leishmania* spp. were in the range reported for this drug as well as the cytotoxicity against mouse peritoneal macrophages [27, 28].

### 3.3. In Vivo Studies

**Figure 3:**
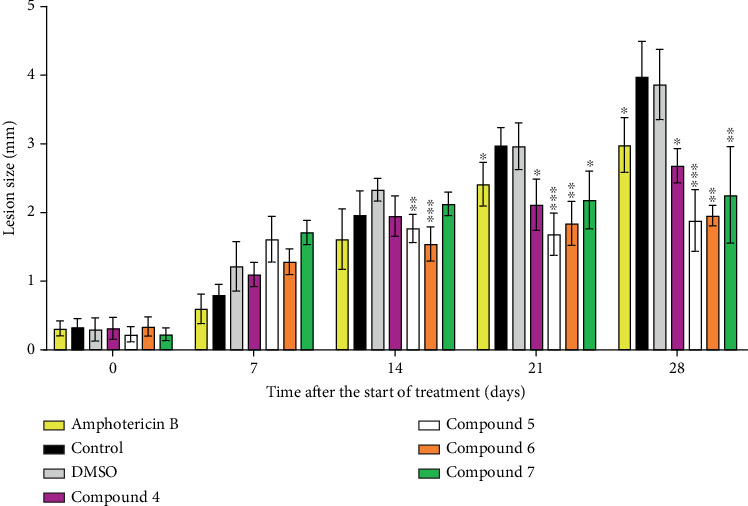
Progression of lesion size since the start of treatment. Mice were infected in the footpads with *L. mexicana* promastigotes and treated for 2 weeks with the test compounds once lesions developed (14 days after infection). The dorso-plantar diameters of the infected (right) and noninfected (left) limbs were measured weekly with a caliper, and the size of the lesion was calculated by difference. Data were compared by repeated measures ANOVA and Tukey's test. Statistical significance of amphotericin B versus controls and compounds versus DMSO are depicted. ⁣^∗^*p* < 0.05, ⁣^∗∗^*p* < 0.01, and ⁣^∗∗∗^*p* < 0.001.

Because of the small amount (7.3–20.7 mg) of compounds purchased, due to limited availability in stock of some of them and the price of others, it was not possible to test the compounds by either the oral or intraperitoneal routes. Instead, they were administered intralesionally, dissolved in DMSO. This solvent has very low systemic toxicity (50% lethal single dose toxicity by subcutaneous route of 13.9–20.5 g/kg) [29], but it is locally irritant at high concentration. As expected, some degree of solvent-associated inflammation occurred during the period of administration (observable at Day 7th and 14th of follow-up, Figure 3), which disappeared once treatment was completed. A similar effect was observed in mice treated with the test compounds, which can be reasonably attributed to the solvent. However, 1 and 2 weeks after the end of treatment, all four test compounds showed a statistically significant (*p* < 0.05) reduction of lesion size compared to DMSO (solvent) and negative control groups. Lesion size of mice treated with Compound 5 at 21 days (*p* = 0.0491), and Compounds 5 (*p* = 0.0057) and 6 (*p* = 0.0030) at 21 days were also statistically smaller than those of amphotericin B–treated mice.

**Figure 4:**
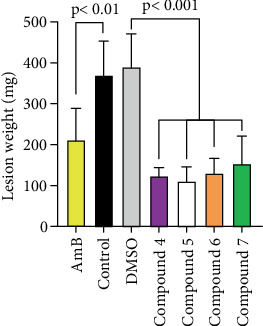
Lesion's weight 2 weeks after the end of treatment. Infected mice were treated for 2 weeks, and 2 weeks later, they were humanely sacrificed, and both rear limbs were excised at the level of the tibiotarsal joint and weighted to calculate, by difference, the weight of the lesions. Data were compared by ANOVA and Tukey's test. Statistical significance of amphotericin B versus controls and compounds versus DMSO are depicted.

At later stages of disease progression, lesion weight is a better estimate of the volume of infected tissue than dorso-plantar diameter, since some lesions tend to be wider or longer than others. Nevertheless, similar results were obtained in terms of lesion weight (Figure 4) as those previously seen for lesion size: the lesion weight of mice treated with any of the test compounds was statistically lower (*p* < 0.001) than those treated with solvent or the control group. Besides, the average lesion weight of mice treated with Compound 5 was statistically lower (*p* = 0.023) than that of amphotericin B treated ones.

**Figure 5:**
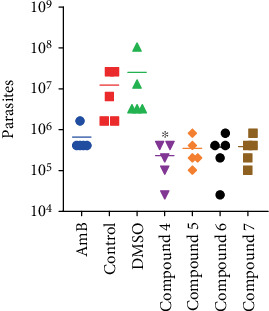
Parasite load 2 weeks after the end of treatment. Mice were infected in the footpads with *L. mexicana* promastigotes and treated by intralesional route for 2 weeks with the test compounds once lesions developed. Mice were sacrificed 2 weeks after the end of treatment, the lesions were excised, and the number of parasites estimated by limiting dilutions. Data were compared by Kruskal-Wallis's test and the distribution-free multiple comparisons test. ⁣^∗^Statistical significance compared to control mice (*p* < 0.05).

Apparently, the number of parasites in the infected tissue (Figure 5) was also reduced in mice treated with the test compounds (*p* = 0.0007, Kruskal–Wallis's test) compared to the control and DMSO groups. However, the distribution-free multiple comparison test only demonstrated statistically significant differences between the number of parasites in mice treated with Compound 4 and controls (*p* = 0.0170), probably due to the number of groups, sample size, and data variability. Paired comparisons (Mann–Whitney test), although not technically the most appropriate procedure, did prove statistical differences (*p* < 0.01) between the samples from mice treated with any of the test compounds and controls or DMSO treated mice. In general, the trend shown by the number of parasites in the infected tissue suggested that the reduction of lesion size and lesion weight was primarily a consequence of the antileishmanial activity of the test compounds and not due to an anti-inflammatory effect.

**Figure 6:**
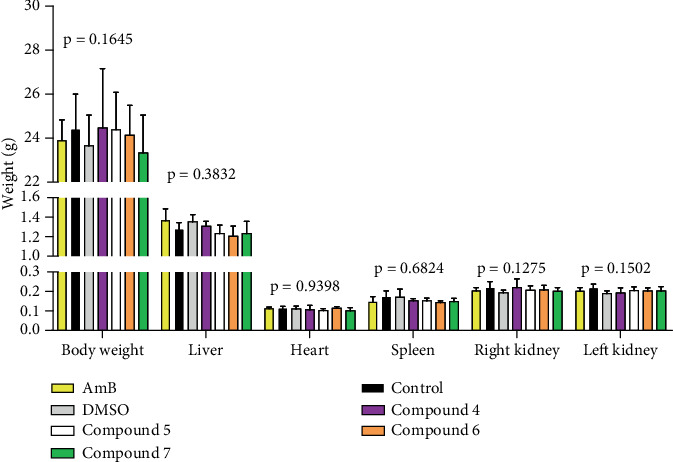
Body and organ weights 2 weeks after the end of treatment. Mice were sacrificed 2 weeks after completing treatment and representative organs were sampled and weighted. *p* values indicate the statistical difference among all the groups (ANOVA) for each of the variables.

Body and organ weights (Figure 6) were not affected by the test compounds (*p* = 0.7421). Mice received an average cumulative dose of about 45 mg/kg (five doses, 10 mg/mL, 0.02 mL, 21.52 g average body weight at the start of treatment) in a regimen of about 9 mg/kg every 72 h. These results preliminarily support the safety of the test compounds at the schedule of administration used.

Sharlow et al. [4] were the first to report the activity of hundreds of thousands of compounds tested by HTS technology against *Leishmania* spp. and posted primary data for public access in PubChem Bioassays. Years later, Khare et al. [7] and Peña et al. [8] reported testing millions of compounds from the collections of Novartis (2.9 million compounds) and GlaxoSmithKline (1.8 million compounds), respectively; although, to our knowledge, the primary data are not publicly available. Instead, summaries of representative compounds considered more promising have been disclosed. Unfortunately, this prevents the research community from exploring the results from other perspectives and limits the possibilities for the development of structure-activity models to predict the antileishmanial activity of new, nontested chemical entities.

Ideally, all the 17,620 active compounds identified in the primary screening at 10 *μ*M (AID 1063) by Sharlow et al. [4] should have been tested at lower concentrations to explore their full potential as antileishmanial agents. Unfortunately, only 1121 compounds were tested at 1 *μ*M [11], of which eventually 93 had IC_50_ values under 1 *μ*M [4].

After near 15 years, AID 1063 data have been poorly exploited. Only two out of the 77 citations (until September 2023) of Sharlow et al.'s paper refer to further testing the compounds previously reported by them as active. Worth mentioning, Zhu et al. [30] assessed 31 out of the 70 compounds tested by Sharlow et al. [4] with IC_50_ values below 1 *μ*M that were also not toxic to mammalian epithelial cancer cells at such concentration. They also tested some structurally related compounds to those found selectively active against *L. donovani* and *L. amazonensis* intracellular amastigotes and eventually identified benzothiazole cyanines as a scaffold worthy of lead optimization efforts. On that basis, years later, Abdelhameed [31] designed and synthesized cyanines-azole hybrids as an effort to develop new antileishmanial agents.

In the present paper, the results of Sharlow et al. were analyzed from a different viewpoint. Sharlow et al.'s primary objective was identifying novel chemical scaffolds associated with antileishmanial activity. On the contrary, we focused on finding new compounds (structurally different from the ones identified by Sharlow et al.'s Tanimoto coefficients (Tc) from 0.37 to 0.50 and Zhu et al.'s Tc from 0.45 to 0.71) which were not tested at 1 *μ*M and, theoretically, had proper biopharmaceutical properties as well as selective antileishmanial activity.

**Figure 7:**
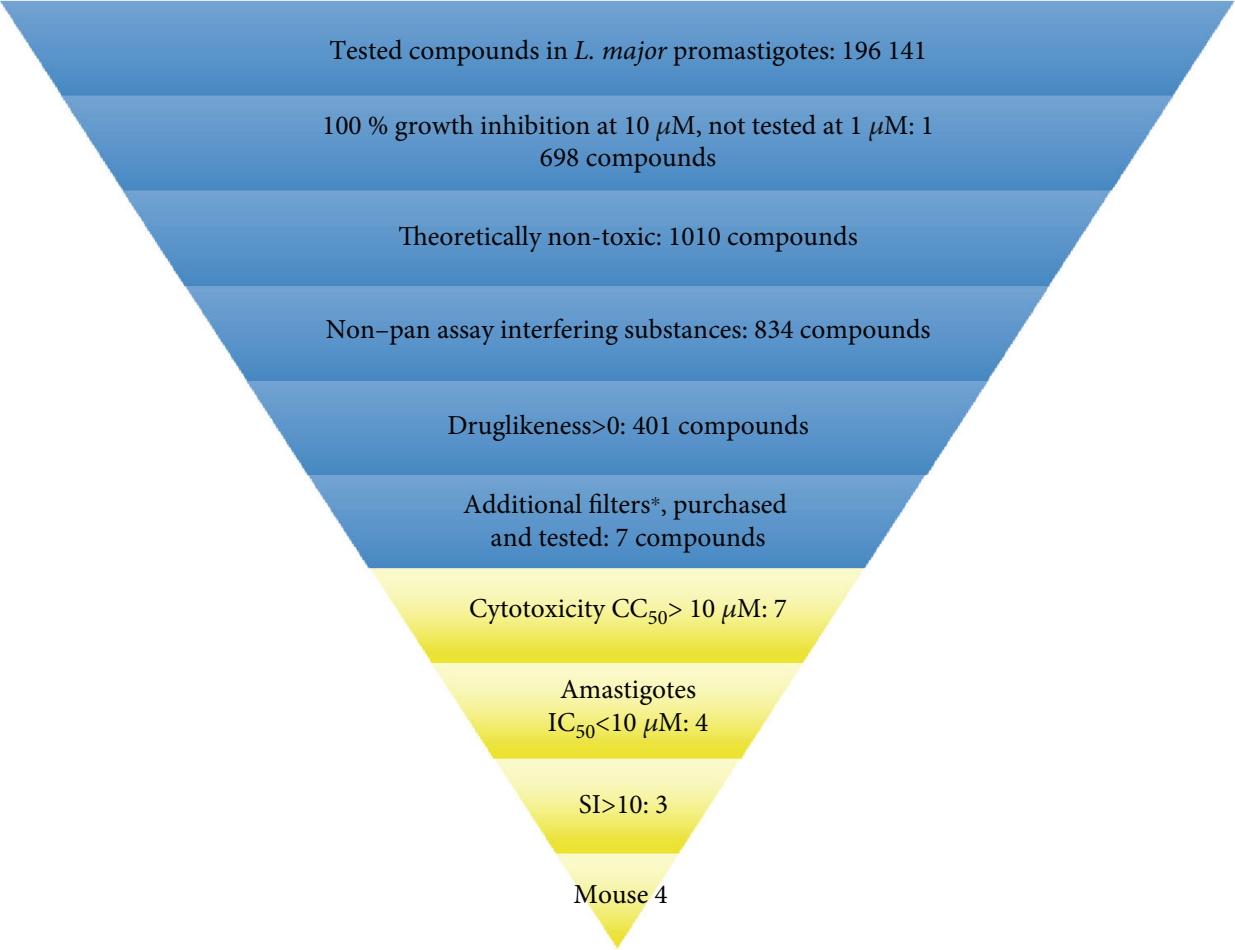
Summary of the filtering process (in blue) and outcome of in vitro and in vivo testing (in yellow) of the selected test compounds. ⁣^∗^Additional filters included low cytotoxicity (CC_50_ > 10* μ*M), positivity rate in PubChem Bioassays under 5%, commercial availability, being structurally different from each other, and not having been previously tested against *Leishmania* spp., except in AID 1063. SI, selectivity index.

The correlation of outcomes at 10 and 1 *μ*M led to a simple but eventually efficient discrimination rule. Selecting 100% growth inhibiting compounds at 10 *μ*M resulted in four out of seven compounds that were selectively active against intracellular *L. mexicana* amastigotes and preliminarily active and safe in a model of experimental cutaneous leishmaniasis (Figure 7). Overall, the results presented here support the potential of these four compounds as antileishmanials and the great potential of the primary data published in the bioassay AID 1603 as a source of new antileishmanial agents.

Phenotypic screening (like the ones used in the present paper), as opposed to target-based screening, does not require prior knowledge of the molecular target; instead, it identifies compounds based on their ability to produce phenotypic effects, such as growth inhibition and cell death. It can reveal unexpected mechanisms of action, including those that involve multiple targets or pathways. Besides, phenotypic screening methods are more likely to discover compounds with novel modes of action since they are not limited to predefined targets. However, they cannot reveal the mode of action of identified hits. On this basis, regarding the compounds found in the present paper with antileishmanial potential, it is not possible to speculate on specific molecular targets supporting their mechanism of action.

Over the years, a wealth of results from large-scale in vitro screening campaigns has accumulated (Siqueira-Neto et al., 2010; [6–8, 32, 33]); therefore, it is likely that the chemical space has already been sufficiently explored in vitro, and a number of promising chemical scaffolds have been proposed. Hence, we consider it is time for the research community to take advantage of all available information to move forward with previously tested compounds rather than testing new ones, so that previous efforts and expenditures are translated into societal impact.

## Figures and Tables

**Table 1:** Toxicity estimates by using Osiris DataWarrior software.

**Level of toxicity**	**Mutagenic**	**Tumorigenic**	**Reproduction effects**	**Irritation**
None	1415	1447	1549	1534
Low	80	52	75	85
High	351	347	222	227

**Table 2:** Molecular properties of the final set of compounds.

**Compound**	**PubChem ID**	**Molecular weight**	**MLOGP**	**H-acceptors**	**H-donors**	**Druglikeness**	**Drugscore**
1	417352	271.4	2.39	3	1	3.994	0.8508
2	706914	264.4	1.99	3	0	6.010	0.9258
3	314212	194.3	1.78	3	1	1.432	0.8620
4	4362222	298.5	3.64	2	0	8.415	0.8587
5	662265	371.5	2.63	7	2	4.302	0.8515
6	15945681	191.2	1.76	3	1	2.525	0.9314
7	3103717	360.3	2.91	**11**	0	2.026	0.8374
Lipinski's rule of five		≤ 500	≤ 4.15	≤ 10	≤ 5		

*Note:* Underlined texts contained in the manuscript hyperlink to detailed information of the compound in PubChem. Bold text highlights the value for the only compound that did not fulfill the rule of ≤H-acceptors. Thus, it is relevant.

Abbreviation: MLOGP, Moriguchi octanol-water partition coefficient.

**Table 3:** In vitro activity and cytotoxicity of the test compounds.

**Compound**	**Promastigotes (** **I** **C**_50_ ± **SD)**				**Cytotoxicity** **(** **C** **C**_50_ ± **SD)**	***L. mexicana* amastigotes** **(** **I** **C**_50_ ± **SD)**	**Selectivity index**
	***L. mexicana***	***L. infantum***	***L. major***	***L. amazonensis***			
1	> 50	23.4 ± 9.2	42.5 ± 7.2	> 50	125 ± 11	26.3 ± 8.2	5
2	> 50	> 50	> 50	> 50	127 ± 2.1	> 50	—
3	> 50	> 50	> 50	30.6 ± 11	> 257	> 50	—
4	11.1 ± 8.2	23.1 ± 7.6	11.3 ± 2.2	23.7 ± 9.9	172 ± 6.9	8.9 ± 1.5	19
5	27.2 ± 6.2	13.8 ± 4.1	9.9 ± 1.9	28.6 ± 14.3	172 ± 7.9	1.1 ± 0.3	157
6	2.3 ± 0.54	2.7 ± 1.0	4.6 ± 2.3	3.0 ± 0.42	60.9 ± 10.7	5.9 ± 2.8	10
7	0.76 ± 0.16	5.9 ± 1.0	2.9 ± 0.56	1.2 ± 0.44	25.2 ± 1.9	0.23 ± 0.04	110
AmB	0.042 ± 0.019	0.031 ± 0.002	0.031 ± 0.008	0.031 ± 0.007	21.0 ± 2.1	0.48 ± 0.02	44

*Note:* All values are expressed in micromolar. Selectivity index = CC_50_/IC_50(in amastigotes)_.

Abbreviations: AmB, amphotericin B deoxycholate; SD, standard deviation.

## Data Availability

Primary data is available in Sifontes-Rodríguez et al. [17, 20, 34], “PubChem BioAssays 1063: A Poorly Exploited Source of New Antileishmanial Compounds” (dataset), Zenodo. 10.5281/zenodo.10084616.
